# Effects of High Sugar Content on Fermentation Dynamics and Some Metabolites of Wine-Related Yeast Species *Saccharomyces cerevisiae*, *S. uvarum* and *Starmerella bacillaris*

**DOI:** 10.17113/ftb.58.01.20.6461

**Published:** 2020-03

**Authors:** Borbála Oláhné Horváth, Diána Nyitrainé Sárdy, Nikolett Kellner, Ildikó Magyar

**Affiliations:** Szent István University Faculty of Horticultural Science Department of Oenology, Ménesi út 45, 1118 Budapest, Hungary

**Keywords:** *Starmerella bacillaris*, *Candida zemplinina*, non-*Saccharomyces* yeast, high sugar concentration, metabolic footprint

## Abstract

*Starmerella bacillaris* (synonym *Candida zemplinina*) is an important non-S*accharomyces* yeast in winemaking with valuable oenological properties, accompanying *Saccharomyces* species in sweet wine fermentation, and has been suggested also for application as combined starter culture in dry or sweet wines. In this study, the major metabolites and nitrogen utilization of these yeasts are evaluated in the musts with high or extremely high sugar concentration. The change in the metabolic footprint of *Saccharomyces cerevisiae*, *Saccharomyces uvarum* and *Starmerella bacillaris* strains was compared when they were present as pure cultures in chemically defined grape juice medium with 220 and 320 g/L of sugar, to represent a fully matured and an overripe grape. Surprisingly, the extreme sugar concentration did not result in a considerable change in the rate of sugar consumption; only a shift of the sugar consumption curves could be noticed for all species, especially for *Starmerella bacillaris*. At the extreme sugar concentration, *Starmerella bacillaris* showed excellent glycerol production, moderate nitrogen demand together with a noticeable proline utilisation. The change in the overall metabolite pattern of *Starmerella bacillaris* allowed clear discrimination from the change of the *Saccharomyces* species. In this experiment, the adequacy of this non-*Saccharomyces* yeast for co-fermentation in juices with high sugar concentration is highlighted. Moreover, the results suggest that *Starmerella bacillaris* has a more active adaptation mechanism to extremely high sugar concentration.

## INTRODUCTION

Fermentation of natural sweet wines has always been a big challenge in winemaking, but the global changes in the climate influence the parameters of the raw grape juice, and as a consequence the quality of the dry or semi-sweet wines. The higher initial sugar concentration is one of the most important factors involved, which could be a considerable challenge for the yeasts. Moreover, not only the fermentation dynamics and ethanol concentration but also the whole metabolite-profile could be modified remarkably (*1*, *2*). In making some special sweet wines like botrytized or straw wines, sugar content of the grapes may reach an extremely high level, multiplying these effects.

Using starter cultures for wine fermentation originally meant exclusively single-strain (*Saccharomyces cerevisiae* or occasionally *Saccharomyces uvarum*) products. During the last decade, the industrial exploitation of selected non-*Saccharomyces* yeasts in combination with *Saccharomyces* strains as oligo-starter cultures has become an emerging trend (*3*-*5*). One of the promising candidates is *Starmerella bacillaris* (syn. *Candida zemplinina*) (*2*).

*S. bacillaris* was originally described in Tokaj wine region in Hungary (*6*) as *Candida zemplinina* and renamed by Duarte *et al.* (*7*). This species is particularly associated with sweet, botrytized wine fermentations (*8*, *9*), where it spontaneously co-exists with *S. cerevisiae and S. uvarum,* but it is frequently isolated from other wine-related sources (*10*, *11*). From oenological aspect, this species has several valuable properties like high glycerol production, fructophilic character and outstanding osmotolerance, reviewed by Englezos *et al.* (*12*).

The major metabolite pattern, in terms of particular fermentation by-products, plays a significant role in forming the final wine quality, while a moderate nitrogen demand, osmotolerance and fructophilic behaviour could help to avoid sluggish or stuck fermentation. These properties are highly varying on interspecific level, although there could be a considerable difference on intraspecific level too. The conventional wine yeasts, *S. cerevisiae* and *S. uvarum* are well-characterized from this aspect (*13*, *14*), but *S. bacillaris* along with other non-conventional yeasts have only recently been in the focus of wine research (e.g. *15**,**16*). Going one step further, a relevant question is how an extreme sugar concentration can modify the above-described character of different wine yeasts.

The aim of this study is to compare and contrast the changes in some major metabolite products and nitrogen utilization induced by extremely high (320 g/L) initial sugar concentration with a normal, but still considerable (220 g/L), sugar level, of the two most important wine yeasts *Saccharomyces cerevisiae* and *Saccharomyces uvarum,* and *Starmerella bacillaris*. Although this last species has been thoroughly studied in recent years at moderate sugar concentrations, to our knowledge, only a few studies investigated high sugar concentrations (*17*-*19*). In our work, we performed a direct comparison among the three species. The metabolites and substrates analysed in this paper were chosen on the basis of their outstanding oenological importance and included the ethanol, glycerol, volatile acidity, l-malic acid, l-succinic acid, yeast assimilable nitrogen and proline. The emphasis was on the effects of extremely high sugar concentration on these non-volatile compounds, but the behaviour of the different yeast species was also compared.

## MATERIALS AND METHODS

### Yeast strains

Strains of three yeast species used in this study are as follows: three *Starmerella bacillaris* strains: Y1667 (CBS9494 type strain) and Y1756 from the National Collection of Agricultural and Industrial Microorganisms (NCAIM), Budapest, Hungary, isolated from botrytized Tokaj grape, and strain MLO from the Department of Oenology, Szent István University (DO-SZIU) culture collection, Budapest, Hungary, isolated from wine, six *Saccharomyces cerevisiae* strains: UVAFERMPM and UVAFERM228 commercial wine yeasts, strains SC57, RA100 and SB12 from DO-SZIU, isolated from Tokaj Aszú wine, and strain S701 also from DO-SZIU, isolated from the wine from Somló wine region, and three *Saccharomyces uvarum* strains: CBS395^T^, type strain from NCAIM, SB42 from DO-SZIU, both isolated from Tokaj Aszú wine, and S103 from DO-SZIU, isolated from the wine from Somló wine region. The natural isolates were previously identified at the Department of Microbiology and Biotechnology, Szent István University (*20*).

### Culture media

The fermentation was carried out in chemically defined grape juice medium at 220 and 320 g/L of sugar. Based on Henschke and Jiranek (*21*), the medium included (per litre of distilled water) d-glucose 110 or 160 g, d-fructose 110 or 160 g, KOH 4 mL to adjust pH to 3.3, yeast carbon base 11.7 g, Tween 80 0.5 mL, l-tartaric acid 2 g and l-malic acid 3 g. To obtain the yeast assimilable nitrogen (YAN) level of 300 mg/L, the following mixture of single amino acids and diammonium phosphate was used (in mg/L in the final medium): arginine 657.0, asparagine 131.0, aspartic acid 263.0, glutamine 175.0, glutamic acid 438.0, histidine 8.8, isoleucine 20.5, leucine 67.0, methionine 44.0, phenylalanine 17.5, threonine 43.9, tryptophan 17.5, tyrosine 17.5, valine 87.7 and proline 438.5, which is not part of YAN but present in every grape juice, and diammonium phosphate 136.0. All chemicals were obtained from Sigma-Aldrich Chemie GmbH, Merck, Munich, Germany.

### Yeast culture maintenance

Yeast cultures were maintained on YEPD (20 g/L glucose, 10 g/L peptone, 10 g/L yeast extract and 15 g/L agar-agar) agar slants and stored at 6 °C. For inoculum preparation, an inoculation loop of cells from fresh agar slants was transferred into 20 mL of YEPD broth in 100-mL flasks and incubated at 25 °C for 48 h without agitation.

### Fermentation conditions

Fermentations were carried out under semi-anaerobic conditions, in 200-mL flasks containing 180-mL aliquots of culture media, without shaking, in triplicate. The flasks were inoculated with 10^6^ cell/mL with 3% of 48-hour-old yeast cultures grown in YEPD broth. The fermentation temperature was kept at 20 °C. The course of fermentation was monitored by measuring the total soluble solids content (Brix values) with an Atago RX-5000 CX digital refractometer (Atago Co. Ltd., Fukaya-shi, Japan) after filtration, by sampling on days 0, 2, 4, 7, 9, 14, 21 and 28. Biomass concentration was measured by Bürker chamber cell counting after methylene blue staining. After the monitoring period of 28 days, the samples for chemical analysis were membrane filtered (0.45 μm) and stored at  -18 °C until analysis.

### Chemical and statistical analysis

Ethanol concentrations were measured by distillation and determination of specific gravity of the distillate (*22*), volatile acidity (expressed as acetic acid) by steam distillation followed by titration (*23*) and reducing sugars by iodometric titration (*24*) using the official methods of the International Organisation of Vine and Wine. d-glucose/d-fructose ratio (K-FRUGL), glycerol (K-GCROL), l-succinic acid (K-SUCC), l-malic acid (K-LMAL) and YAN (K-LARGE and K-PANOPA) concentrations were measured with Megazyme enzymatic kits (Bray, Ireland). Proline concentrations were measured by ^1^H NMR technique on a Bruker AVANCE 400 spectrometer and 400’54 ASCEND magnet system (Bruker, Karlsruhe, Germany) (*25*).

Data were evaluated with analysis of variance one way (ANOVA) and multivariate (MANOVA) and discriminant analysis (DA), after checking the assumptions, using the statistical package IBM SPSS Statistics for Windows v. 23.0 (*26*).

## RESULTS AND DISCUSSION

### Sugar consumption dynamics

**Fig. 1:**
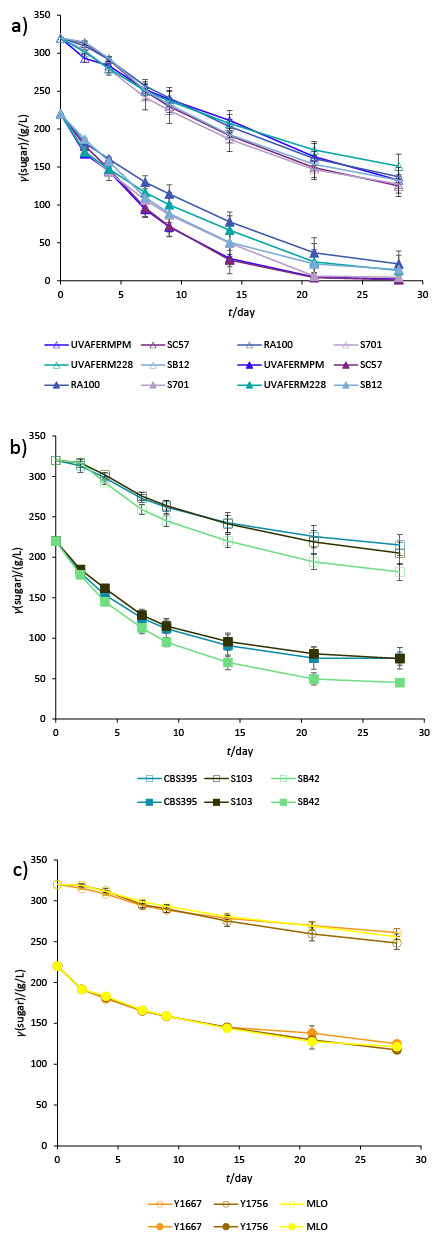
Sugar consumption dynamics of the investigated strains in chemically defined grape juice: a) *S. cerevisiae*, b) *S. uvarum*, and c) *S. bacillaris.* Full symbols: initial *γ*(sugar)=220 g/L, empty symbols: initial *γ*(sugar)=320 g/L. Data are mean values of the triplicate fermentations with standard deviations (*N*=3)

Sugar consumption curves of the different species are shown in Fig. 1. At the end of the monitored period of fermentation with the lower initial sugar concentration (220 g/L), only *S. cerevisiae* strains UVAFERMPM, SC57 and S701 completed the fermentation. After fermentation with S. *cerevisiae* strains RA100, UVAFERM228 and SB12, smaller amounts of sugar remained (13.6-22.2 g/L), while with all the *S. uvarum* and *S. bacillaris* strains, considerable amounts of sugar remained (45.1-75.3 g/L and 117.5-125.0 g/L respectively), which is in accordance with the earlier described behaviour of the given species (*9*).

The most striking observation is that the extreme sugar concentration (320 g/L) did not influence significantly the rate of sugar consumption by *S. uvarum*, and in particular by *S. bacillaris* strains. Only a shift can be noticed in the sugar consumption curves, according to the elevated initial sugar content of the juice, resulting in higher residual sugar concentrations (Fig. 1).

### Effect of extreme sugar concentration on the major metabolites

Our focus was on the major by-products of the alcoholic fermentation present in the chemically defined grape juice at the end of the monitored period (28 days) (Table 1). This experiment confirmed the earlier described strong fructophilic nature of *S. bacillaris* (*8*, *9*) (Table 1), and this characteristic was not influenced by the high sugar concentration, therefore, it was excluded from further analysis and the sum of the consumed glucose and fructose was used for the yields, if applicable (Table 2 and Table 3).

**Table 1:** Major metabolites and cell density of chemically defined grape juice fermented with different yeast species at initial *γ*(sugar)=220 and 320 g/L

*γ*(sugar)/(g/L)	Species	Strain	*φ*(ethanol)/%	*γ*(glycerol)/(g/L)	*γ*(acetic acid)/(g/L)	*γ*(consumed malic acid)/(g/L)	*γ*(succinic acid)/(g/L)	Glucose-fructose ratio	*N*·10^8^/(cell/mL)
220	*S. cerevisiae*	UVAFERMPM	(12.9±0.2)^d^	(4.7±0.3)^a^*	(0.72±0.01)^c^*	(0.7±0.1)^abcd^	(0.44±0.01)^c^*	(0.3±0.2)^a^	(2.7±1.2)^ab^
		SC57	(12.8±0.2)^d^	(4.7±0.5)^a^*	(0.68±0.07)^bcd^*	(0.6±0.2^ab^	(0.32±0.01)^b^	(0.8±0.4)^a^	(2.7±0.3)^a^
		S701	(12.6±0.2)^cd^*	(5.1±0.3)^ab^*	(0.78±0.02)^c^*	(0.6±0.1)^abc^	(0.31±0.02)^ab^	(0.08±0.01)^a^	(2.0±0.6)^ab^
		RA100	(11.2±2.0)^cd^	(5.0±0.3)^ab^*	(0.76±0.05)^cd^*	(0.3±0.1)^a^	(0.25±0.05)^b^	(0.4±0.2)^a^	(1.19±0.07)^ab^
		UVAFERM228	(11.6±1.3)^cd^	(4.9±0.8)^a^*	(0.70±0.06)^cd^*	(0.49±0.06)^ab^	(0.29±0.04)^ab^	(0.18±0.01)^a^	(2.2±0.3)^ab^
		SB12	(13.1±0.2)^cd^	(5.45±0.07)^ab^*	(1.00±0.00)^d^*	(0.46±0.00)^a^	(0.31±0.02)^a^	(0.5±0.3)^a^	(1.4±0.5)^ab^
		**Mean**	**(12.4±1.4)**	**(5.0±0.5)**	**(0.8±0.1)**	**(0.5±0.2)**	**(0.32±0.07)**	**(0.4±0.3)**	**(2.0±0.7)**
	*S. uvarum*	CBS395	(7.6±0.7)^bc^	(5.8±0.4)^ab^*	(0.52±0.03)^b^*	(0.73±0.06)^bcd^*	(0.31±0.03)^b^*	(0.39±0.07)^a^	(1.2±0.2)^b^
		S103	(7.6±0.4)^bc^	(6.5±0.8)^abc^*	(0.46±0.03)^b^*	(0.59±0.06)^abc^	(0.26±0.05)^ab^	(0.39±0.04)^a^	(1.74±0.09)^ab^
		SB42	(9.9±0.5)^bc^	(5.4±0.4)^ab^*	(0.28±0.01)^a^*	(0.73±0.06)^bcd^*	(0.27±0.04)^ab^	(0.20±0.02)^a^	(1.7±0.2)^ab^
		**Mean**	**(8.4±1.2)**	**(5.9±0.7)**	**(0.4±0.1)**	**(0.68±0.09)**	**(0.28±0.04)**	**(0.3±0.1)**	**(1.5±0.3)**
	*S. bacillaris*	Y1667	(4.4±0.2)^a^	(7.8±0.7)^bc^*	(0.8±0.1)^cd^	(0.9±0.1)^cde^	(0.35±0.02)^ab^*	(4.9±1.1)^b^	(2.1±0.5)^ab^
		Y1756	(4.8±0.3)^a^	(7.8±0.2)^bc^*	(0.74±0.08)^c^*	(1.09±0.06)^e^*	(0.40±0.03)^ab^	(5.8±1.4)^b^	(2.0±05)^ab^
		MLO	(4.6±0.3)^a^	(7.4±0.6)^c^*	(0.7±0.1)^bcd^*	(0.96±0.1)^de^*	(0.40±0.03)^ab^*	(6.0±1.7)^b^	(2.0±0.2)^ab^
		**Mean**	**(4.6±0.3)**	**(7.6±0.5)**	**(0.7±0.1)**	**(1.0±0.1)**	**(0.39±0.03)**	**(5.6±1.3)**	**(2.0±0.4)**
	LSD_5%S.c-other_		1.0	0.45	0.09	0.11	n.s.	0.57	n.s.
	LSD_5%S.u.-S.b._		1.1	0.52	0.11	0.13	n.s.	0.65	n.s.
320	*S. cerevisiae*	UVAFERMPM	(11.3±1.3)^BCD^	(9.0±0.3)^A^*	(1.23±0.06)^C^*	(0.53±0.06)^A^	(0.35±0.05)^ABC^*	(0.46±0.05)^A^	(1.2±0.2)^AB^
		SC57	(14.3±1.2)^D^	(8.7±0.2)^A^*	(1.30±0.00)^C^*	(0.4±0.1)^A^	(0.30±0.02)^A^	(0.47±0.03)^A^	(1.8±0.6)^AB^
		S701	(13.6±0.4)^D^*	(9.6±0.4)^A^*	(1.63±0.06)^D^*	(0.49±0.06)^A^	(0.30±0.02)^A^	(0.34±0.03)^A^	(1.7±0.4)^A^
		RA100	(13.2±0.6)^D^	(8.4±0.4)^A^*	(1.3±0.1)^BCD^*	(0.3±0.1)^A^	(0.22±0.00)^AB^	(0.61±0.03)^A^	(1.0±0.2)^AB^
		UVAFERM228	(11.5±2.8)^ABCD^	(8.2±0.6)^A^*	(1.3±0.2)^BCD^*	(0.6±0.2)^A^	(0.27±0.05)^AB^	(0.44±0.09)^A^	(2.4±0.3)^AB^
		SB12	(13.3±0.3)^CD^	(10.6±0.2)^CDE^*	(1.8±0.0)^D^*	(0.43±0.06)^A^	(0.27±0.02)^AB^	(0.41±0.02)^A^	(1.3±0.5)^AB^
		**Mean**	**(12.9±1.6)**	**(9.1±0.9)**	**(1.4±0.2)**	**(0.5±0.1)**	**(0.28±0.05)**	**(0.46±0.09)**	**(1.6±0.6)**
	*S. uvarum*	CBS395	(6.5±1.2)^AB^	(8.3±0.4)^AB^*	(0.86±0.00)^ABC^*	(0.49±0.06)^A^*	(0.21±0.02)^BC^*	(0.69±0.05)^A^	(0.9±0.2)^A^
		S103	(8.1±1.3)^ABC^	(8.6±1.0)^A^*	(0.74±0.01)^A^*	(0.53±0.06)^A^	(0.21±0.03)^AB^	(0.57±0.06)^A^	(1.26±0.07)^A^
		SB42	(10.2±0.8)^BCD^	(9.0±0.8)^AC^*	(0.67±0.02)^A^*	(0.46±0.00)^A^*	(0.26±0.03)^C^	(0.45±0.03)^A^	(2.0±0.4)^AB^
		**Mean**	**(8.3±1.9)**	**(8.7±0.7)**	**(0.75±0.08)**	**(0.49±0.05)**	**(0.23±0.04)**	**(0.57±0.1)**	**(1.4±0.5)**
	*S. bacillaris*	Y1667	(3.9±0.3)^A^	(11.5±0.8)^BDE^*	(1.0±0.1)^C^	(0.7±0.2)^A^	(0.29±0.02)^BC^*	(2.2±0.2)^B^	(2.3±0.4)^AB^
		Y1756	(4.9±0.4)^A^	(12.0±1.5)^BE^*	(1.2±0.3)^BCD^*	(0.7±0.2)^A^*	(0.35±0.05)^C^	(2.8±0.5)^B^	(2.1±0.2)^B^
		MLO	(4.2±0.3)^A^	(10.9±1.0)^BCDE^*	(0.85±0.01)^ABC^*	(0.49±0.06)^A^*	(0.31±0.04)^BC^*	(2.4±0.2)**^B^**	(1.9±0.3)^AB^
		**Mean**	**(4.3±0.5)**	**(11.5±1.1)**	**(1.0±0.2)**	**(0.6±0.2)**	**(0.32±0.04)**	**(2.5±0.4)**	**(2.1±0.3)**
	LSD_5%S.c-other_		1.2	0.75	0.17	n.s.	n.s.	0.17	n.s.
	LSD_5%S.u.-S.b._		1.4	0.87	0.20	n.s.	n.s.	0.19	n.s.

Values are mean of triplicate fermentations for each strain, bold values are species mean values of strain performance. According to the Games-Howell *post hoc* comparison test, the values in the same column are statistically different at p<0.05; different lower-case letters indicate difference at *γ*(sugar)=220 g/L, capital letters at *γ*(sugar)=320 g/L, and asterisks denotes the change induced by initial sugar concentration in the grape. LSD_5%_ values are the least significant differences between the mean values of species: LSD_5%S.c-other_ between *S. cerevisiae* and the other two species, and LSD_5%S.u.-S.b._ between *S. uvarum* and *S. bacillaris*. n.s.=not significant

**Table 2:** Yields of the main fermentation products calculated from the major metabolites of the different yeast species in chemically defined grape juice at initial *γ*(sugar)= 220 and 320 g/L

*γ*(sugar)/(g/L)	Species	Strain	*Y*(ethanol)/(g/g)	*m*(sugar)/g	*Y*(glycerol)/(g/g)	*Y*(glycerol/ ethanol)/(g/g)	*Y*(acetic acid)/(mg/g)	*Y*(succinic acid)/(g/g)
220	*S. cerevisiae*	UVAFERMPM	(0.47±0.01)^c^	(16.9±0.2)^a^	(0.02±0.00)^a^*	(0.05±0.00)^a^*	(3.29±0.04)^b^	(2.01±0.02)^b^*
		SC57	(0.46±0.01)^c^	(17.1±0.3)^a^	(0.02±0.00)^a^*	(0.05±0.00)^a^*	(3.1±0.3)^bc^	(1.47±0.04)^a^
		S701	(0.46±0.01)^bc^	(17.1±0.3)^a^	(0.02±0.00)^a^*	(0.05±0.01)^a^*	(3.7±0.1)^bc^*	(1.6±0.1)^ab^
		RA100	(0.45±0.07)^c^	(17.7±3.0)^a^	(0.03±0.00)^ab^*	(0.06±0.01)^ab^*	(3,9±0.3)^b^	(1.4±0.2)^ab^
		UVAFERM228	(0.44±0.04)^bc^	(18.2±1.9)^a^	(0.02±0.00)^a^*	(0.05±0.00)^ab^*	(3.4±0.3)^b^*	(1.4±0.2)^a^
		SB12	(0.47±0.01)^c^	(16.8±0.2)^a^	(0.03±0.00)^a^*	(0.05±0.00)^ab^*	(4.58±0.01)^c^*	(1.4±0.1)^a^*
		**Mean**	**(0.46±0.03)**	**(17.3±1.3)**	**(0.02±0.00)**	**(0.05±0.01)**	**(3.7±0.9)**	**(1.6±0.3)**
	*S. uvarum*	CBS395	(0.42±0.01)^b^	(18.9±0.4)^a^	(0.04±0.00)^c^*	(0.10±0.00)^c^*	(3.6±0.1)^bc^*	(2.15±0.01)^b^*
		S103	(0.42±0.01)^bc^	(18.8±0.7)^a^	(0.05±0.00)^c^*	(0.11±0.01)^c^*	(3.2±0.2)^b^	(1.8±0.3)^ab^
		SB42	(0.45±0.02)^bc^	(17.8±0.8)^a^	(0.03±0.00)^b^*	(0.07±0.00)^b^*	(1.61±0.05)^a^	(1.5±0.2)^ab^
		**Mean**	**(0.43±0.02)**	**(18.5±0.8)**	**(0.04±0.01)**	**(0.09±0.02)**	**(2.8±0.9)**	**(1.8±0.3)**
	*S. bacillaris*	Y1667	(0.34±0.01)^a^*	(23.1±0.4)^b^*	(0.08±0.00)^d^*	(0.23±0.02)^d^*	(7.8±1.1)^e^	(3.50±0.06)^c^*
		Y1756	(0.35±0.01)^a^	(22.8±0.9)^b^	(0.07±0.00)^d^*	(0.21±0.01)^d^*	(6.7±0.6)^de^	(3.7±0.2)^c^*
		MLO	(0.34±0.02)^a^*	(22.9±1.1)^b^*	(0.07±0.00)^d^*	(0.20±0.00)^d^*	(6.3±0.9)^d^	(3.8±0.2)^c^*
		**Mean**	**(0.34±0.01)**	**(23.0±0.8)**	**(0.07±0.00)**	**(0.21±0.01)**	**(6.9±1.0)**	**(3.7±0.2)**
	LSD_5%S.c-other_		0.02	0.9	0.01	0.01	0.76	0.21
	LSD_5%S.u.-S.b._		0.02	1.1	0.01	0.01	0.88	0.24
320	*S. cerevisae*	UVAFERMPM	(0.45±0.01)^A^	(17.5±0.5)^AB^	(0.05±0.00)^A^*	(0.10±0.01)^AB^*	(6.3±0.3)^BCD^	(1.8±0.1)^CD^*
		SC57	(0.49±0.01)^A^	(16.3±0.4)^A^	(0.04±0.00)^A^*	(0.08±0.01)^A^*	(5.6±0.4)^BC^	(1.28±0.03)^ABC^
		S701	(0.46±0.01)^A^	(17.1±0.2)^AB^	(0.04±0.00)^A^*	(0.09±0.00)^A^*	(7.1±0.3)^CD^*	(1.29±0.08)^AB^
		RA100	(0.47±0.01)^A^	(16.7±0.5)^A^	(0.04±0.00)^A^*	(0.08±0.01)^A^*	(6.0±0.8)^BCD^	(1.00±0.07)^A^
		UVAFERM228	(0.5±0.1)^A^	(17.3±3.6)^AB^	(0.04±0.00)^A^*	(0.09±0.02)^AB^*	(6.7±0.9)^BCD^*	(1.4±0.2)^AB^
		SB12	(0.46±0.00)^AB^	(17.1±0.1)^AB^	(0.05±0.00)^AB^*	(0.10±0.00)^AB^*	(8.0±0.1)^DE^*	(1.20±0.06)^AB^*
		**Mean**	**(0.47±0.04)**	**(17.0±1.3)**	**(0.04±0.00)**	**(0.09±0.01)**	**(6.6±0.9)**	**(1.3±0.3)**
	*S. uvarum*	CBS395	(0.38±0.05)^C^	(21.3±2.7)^B^	(0.06±0.00)^C^*	(0.17±0.03)^B^*	(6.3±0.4)^BCD^*	(1.6±0.1)^BC^*
		S103	(0.40±0.03)^BC^	(19.8±1.3)^AB^	(0.05±0.00)^BC^*	(0.14±0.00)^B^*	(4.7±0.5)^AB^	(1.34±0.09)^ABC^
		SB42	(0.45±0.03)^ABC^	(17.4±1.2)^AB^	(0.05±0.00)^ABC^*	(0.11±0.01)^AB^*	(3.8±0.1)^A^	(1.5±0.2)^ABC^
		**Mean**	**(0.41±0.05)**	**(19.5±2.3)**	**(0.06±0.01)**	**(0.14±0.03)**	**(4.9±1.2)**	**(1.5±0.2)**
	*S. bacillaris*	Y1667	(0.27±0.03)^D^*	(29.2±2.6)^C^*	(0.10±0.00)^D^*	(0.38±0.01)^C^*	(9.4±1.4)^E^	(2.6±0.2)^D^*
		Y1756	(0.30±0.03)^D^	(25.3±0.5)^C^	(0.10±0.00)^D^*	(0.33±0.03)^C^*	(9.9±1.5)^E^	(2.8±0.3)^D^*
		MLO	(0.30±0.00)^D^*	(26.2±0.4)^C^*	(0.10±0.00)^D^*	(0.33±0.02)^C^*	(7.6±0.8)^DE^	(2.8±0.2)^D^*
		**Mean**	**(0.29±0.03)**	**(26.9±2.2)**	**(0.10±0.01)**	**(0.34±0.03)**	**(9.0±1.5)**	**(2.7±0.2)**
	LSD_5%S.c-other_		0.03	1.5	0.01	0.02	0.95	0.19
	LSD_5%S.u.-S.b._		0.04	1.8	0.01	0.02	1.09	0.22

Values are mean of triplicate fermentations for each strain, bold values are species mean values of strain performance. After the Games-Howell *post hoc* comparison, the mean values in the same column are statistically different at p<0.05; different lower-case letters indicate difference at *γ*(sugar)=220 g/L, capital letters at *γ*(sugar)=320 g/L, and asterisks denotes the change induced by initial sugar concentration in the grape. LSD_5%_ values are the least significant differences between the mean values of species: LSD_5%S.c-other_ between *S. cerevisiae* and the other two species, and LSD_5%S.u.-S.b._ between *S. uvarum* and *S. bacillaris*. n.s.=not significant

**Table 3:** Some nitrogen-related properties of the different yeast species in chemically defined grape juice at initial *γ*(sugar)=220 and 320 g/L

*γ*(sugar)/(g/L)	Species	Strain	*γ*(consumed YAN)/(mg/L)	*γ*(consumed proline)/(mg/L)	Specific YAN consumption/sugar	Specific YAN consumption/ biomass
220	*S. cerevisiae*	UVAFERMPM	(141.2±13.0)^ab^	N.D.	(0.48±0.06)^a^	(0.52±0.05)^a^*
		SC57	(196.3±4.6)^ab^*	(0.2±0.3)^a^	(0.73±0.02)^bc^*	(0.72±0.02)^bc^
		S701	(122.9±5.2)^c^	N.D.	(0.40±0.02)^a^	(0.61±0.03)^ab^*
		RA100	(191.0±2.5)^b^*	N.D.	(0.79±0.03)^cd^*	(1.60±0.02)^e^
		UVAFERM228	(143.4±13.5)^ab^	N.D.	(0.51±0.07)^ab^	(0.7±0.6)^ab^
		SB12	(129.6±4.7)^c^	N.D.	(0.42±0.02)^a^	(0.95±0.03)^d^
		**Mean**	**(154.1±31.1)**	**(0.03±0.1)**	**(0.6±0.2)**	**(0.8±0.4)**
	*S. uvarum*	CBS395	(211.7±4.4)^d^*	N.D.	(1.23±0.01)^e^*	(1.76±0.04)^e^
		S103	(219.6±2.3)^d^*	N.D.	(1.28±0.08)^e^*	(1.26±0.04)^e^*
		SB42	(139.4±14.2)^ab^	N.D.	(0.59±0.08)^ab^	(0.84±0.01)^cd^*
		**Mean**	**(190.3±39.0)**	**N.D.**	**(1.0±0.4)**	**(1.3±0.2)**
	*S. bacillaris*	Y1667	(138.5±15.7)^a^*	N.D.	(1.0±0.1)^de^*	(0.67±0.08)^b^*
		Y1756	(172.9±2.6)^ab^*	(3.4±3.9)^a^*	(1.24±0.02)^e^*	(0.86±0.08)^d^*
		MLO	(173.1±7.0)^ab^*	(2.5±4.4)^a^*	(1.3±0.01)^e^*	(0.9±0.4)^d^*
		**Mean**	**(161.5±19.3)**	**(2.0±3.3)**	**(1.2±0.2)**	**(0.8±0.1)**
	LSD_5%S.c-other_		n.s	n.s	0.18	n.s.
	LSD_5%S.u.-S.b._		n.s.	n.s.	0.21	n.s.
320	*S. cerevisiae*	UVAFERMPM	(144.7±6.5)^A^	(12.3±6.0)^AB^*	(0.58±0.08)^AB^	(1.21±0.06)^A^*
		SC57	(142.1±4.7)^A^*	N.D.	(0.48±0.01)^ABC^*	(0.81±0.03)^B^
		S701	(125.2±6.2)^B^	(4.0±2.5)^AB^	(0.40±0.04)^A^	(0.74±0.04)^C^*
		RA100	(142.6±3.8)^CD^*	(6.7±6.8)^AB^	(0.50±0.03)^ABC^*	(1.49±0.04)^D^
		UVAFERM228	(136.4±6.9)^CD^	(13.8±0.7)^B^*	(0.54±0.02)^ABC^	(0.58±0.03)^E^
		SB12	(119.8±8.2)^AB^	(0.1±0.2)^A^	(0.39±0.04)^A^	(0.89±0.06)^B^
		**Mean**	**(135.1±11.0)**	**(6.1±6.8)**	**(0.48±0.08)**	**(1.0±0.3)**
	*S. uvarum*	CBS395	(119.0±9.6)^C^*	(9.9±9.7)^AB^*	(0.64±0.03)^C^*	(1.3±0.1)^A^
		S103	(114.5±8.5)^C^*	(0.1±0.3)^A^	(0.52±0.05)^ABC^*	(0.91±0.07)^B^*
		SB42	(126.6±1.3)^CD^	N.D.	(0.53±0.01)^ABC^	(0.65±0.01)^CE^*
		**Mean**	**(120.1±8.3)**	**(3.3±6.9)**	**(0.56±0.06)**	**(0.9±0.3)**
	*S. bacillaris*	Y1667	(95.7±11.0)^D^*	(18.6±7.6)^B^*	(0.6±0.1)^BC^*	(0.42±0.05)^F^*
		Y1756	(88.9±3.4)^D^*	(17.6±4.4)^B^*	(0.46±0.05)^AB^*	(0.42±0.02)^F^*
		MLO	(85.0±12.9)^E^*	(18.3±5.6)^B^*	(0.5±0.1)^ABC^*	(0.44±0.07)^F^*
		**Mean**	**(89.9±9.8)**	**(18.2±8.4)**	**(0.50±0.09)**	**(0.43±0.04)**
	LSD_5%S.c-other_		8.4	6.0	n.s.	n.s.
	LSD_5%S.u.-S.b._		9.7	7.0	n.s.	n.s.

Values are mean of triplicate fermentations for each strain, bold values are species mean values of strain performance. After the Games-Howell *post hoc* comparison, the mean values in the same column are statistically different at p<0.05; different lower-case letters indicate difference at *γ*(sugar)=220 g/L, capital letters at *γ*(sugar)=320 g/L, and asterisks denotes the change induced by initial sugar concentration in the grape. LSD_5%_ values are the least significant differences between the mean values of species: LSD_5%S.c-other_ between *S. cerevisiae* and the other two species, and LSD_5%S.u.-S.b._ between *S. uvarum* and *S. bacillaris*. n.s.=not significant. YAN=yeast assimilable nitrogen

*S. bacillaris* strains used the highest concentration of sugars to produce 1% ethanol, the *Saccharomyces* strains used considerably less (Table 2). Evaluating the change, only *S. cerevisiae* S701 was able to produce slightly increased alcohol yield from the excess sugar. The sugar-induced change in the ethanol yield was only significant when using *S. bacillaris* Y1667 and MLO (reduction from 0.34 to 0.27-0.30 g/g), which implies that these strains changed their main metabolite ratio due to the higher osmotic pressure.

The glycerol yield of *S. bacillaris* strains was considerably higher than of the investigated *Saccharomyces* species at both sugar levels (Table 2). The strong positive effect of the increasing sugar concentration was confirmed previously for the *S. cerevisiae* and *S. uvarum* strains and was verified also for *S. bacillaris* in this study, in accordance with the findings of Rantsiou *et al*. (*18*). The high glycerol production of the *S. bacilllaris* strains is highly valuable and it could shape considerably the body of the wine regardless of the sugar level.

The glycerol/ethanol ratio also showed an alteration in the main balance of the alcoholic fermentation, which was 2 to 4 times higher when using *S. bacillaris* than *Saccharomyces* species and clearly distinct at both sugar levels. Consequently, reduced alcohol content and a fuller wine texture could be reached employing *S. bacillaris* strains, preferably in combination with *Saccharomyces* yeasts due to their limited fermentation ability.

It has long been known that the increasing osmotic stress increases the acetic acid production of *Saccharomyces* species and we found similar trend for S. *bacillaris*. *S. uvarum* strains SB42 and S103 had the smallest increase, while CBS395 was similar to *S. bacillaris* strains Y1667, Y1756 and MLO, followed by *S. cerevisiae* strains UVAFERMPM, SC57, RA100 and UVAFERM228 (Table 1). In contrast to these results, Rantsiou *et al*. (*18*) found a sugar-independent trend in the acetic acid production by *S. bacillaris* that was mainly time-dependent. The absolute amount of acetic acid is comparable with that produced by *Saccharomyces* species, which is in accordance with some earlier results (*9*). Considering the limited fermentation ability of *S. bacillaris*, the acetic acid yield is not problematic since this species is not used in pure culture but in mixed fermentation (*15*, *16*).

All species consumed l-malic acid and produced l-succinic acid, at both sugar concentrations. The effect of sugar concentration on the consumption was significant in the case of *S. uvarum* CBS395 and SB42, and *S. bacillaris* Y1756 and MLO, resulting in a reduction of the consumed malic acid. The negative initial sugar dependence of l-malic acid utilisation is in accordance with an earlier study (*18*). The l-succinic acid production was generally low in oenological terms (usual range 0.5-2.0 g/L (*27*)), but the yields were higher in the case of *S. bacillaris* than that of the other strains at both sugar concentrations (Table 2). l-succinic acid production by *S. bacillaris* Y1667 and MLO, *S. cerevisiae* UVAFERMPM and *S. uvarum* CBS395 seems to be influenced negatively by the initial sugar content. In certain vintages, the capability of a positive net organic acid production of a yeast is highly appreciated.

### Nitrogen utilisation

The nitrogen demand of a certain species is highly important in winemaking, and it is even more highlighted in the case of non-*Saccharomyces* species in mixed fermentation, in terms of competition, balance or synergism (*4*, *28*, *29*). Moreover, in grape juices with high sugar concentration, the appropriate amount of YAN could be crucial to complete the fermentation (*21*).

The composition of the YAN mimicked the grape juice composition. YAN concentration was adjusted to 300 mg/L, well above the necessary level (150-200 mg/L; (*21*)), but still below the optimal concentrations (400-500 mg/L).

The YAN utilization by the strains was varying widely, between 30-63%. At the higher sugar concentration, there was a significant decrease in YAN consumption by *S. cerevisiae* SC57 and RA100, *S. uvarum* CBS395 and S103 and all three *S. bacillaris* strains. Although the YAN consumption is reported to increase at higher sugar concentrations, in our investigations we used extremely high sugar concentration, where the nitrogen metabolism might be repressed to some extent (Table 3). The specific YAN consumption data show (Table 3) that the sugar-induced change in its consumption by *S. bacillaris* strains (0.43-0.81 mg/g) was similar to *S. uvarum* CBS395 (0.59 mg/g) and S103 (0.76 mg/g). The absolute YAN consumption by *S. bacillaris* was comparable with that of *Saccharomyces* species at lower sugar concentration, which corresponds to an earlier experiment (*30*). At high sugar concentration, *S. bacillaris* strains showed a moderate demand for nitrogen. This behaviour could be useful in mixed fermentations in juices where the initial sugar concentration is high or even extremely high.

Proline uptake was practically undetected at 220 g/L sugar, but it was taken up poorly at the higher sugar concentration (Table 3). This trend was prevalent for all the species, but moderate and strongly strain-dependent in the *Saccharomyces* species, involving *S. cerevisiae* UVAFERMPM, UVAFERM228 and *S. uvarum* CBS395. However, there was a more considerable increase in the proline utilization at the higher sugar level in the case of all strains of *S. bacillaris*. Proline, which would be an abundant source of nitrogen in every grape juice, is not or is negligibly utilized by wine yeasts due to an inhibition under anaerobic conditions (*29*, *30*). Our results are consistent with this view at the normal sugar concentration, but an improved proline utilization might function at extreme sugar level, particularly in *S. bacillaris*.

### Multivariate performance assessment

**Fig. S1:**
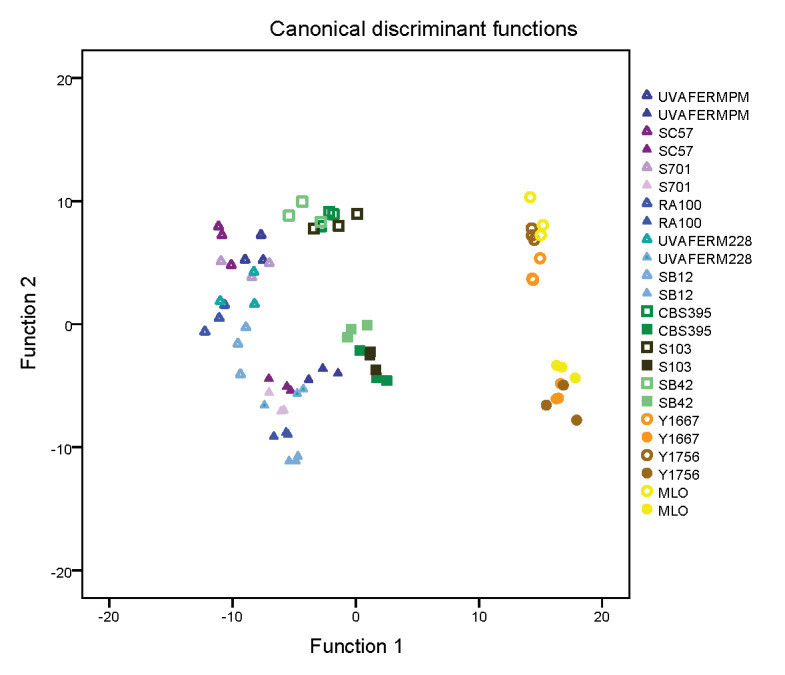
Discriminant function analysis of major metabolites and nitrogen utilization of the different species. *S. cerevisiae* (triangle) *S. uvarum* (square) and *S. bacillaris* (circle) in chemically defined grape juice with initial sugar *γ*=220 (full symbols) and 320 g/L (empty symbols). The analysis classified 95.8% of the cases correctly. Function 1=ethanol volume fraction, function 2=volatile acidity and consumed proline concentrations, and function 3=glycerol, succinic acid, malic acid and consumed yeast assimilable nitrogen concentrations (data not shown)

The sugar-induced changes in the above-described metabolites and nitrogen utilization of the investigated yeasts should be taken into consideration in one model. Fig. S1 shows the results of discriminant function analysis, with three significant functions. The strains were clearly distinct at different sugar concentrations.

Function 1, ethanol, did not group with either factor. Function 2 represented the factors that were sugar-dependent in a positive manner, namely volatile acidity and proline utilization, while Function 3 comprised factors negatively influenced by the sugar level, namely consumption of YAN and l-malic acid, and production of l-succinic acid. The performance of *S. uvarum* strains was overall considerably closer to that of *S. cerevisiae* than of *S. bacillaris* strains. The *S. cerevisiae* strains formed the most scattered group, which indicates their high intraspecies diversity, in terms of their altered metabolite production due to extremely high sugar concentration.

## CONCLUSIONS

The high sugar-driven changes in the major fermentation metabolite pattern and nitrogen utilization pointed out an altered behaviour of the investigated species. The overall performance of *Saccharomyces uvarum* strains was close to *S. cerevisiae* in general; however, with clear differences in certain properties, *e.g*. volatile acid and glycerol production, while *Starmerella bacillaris* strains exhibited distinct, sometimes inverse behaviour than the *Saccharomyces* species. The effects of the extremely high sugar concentration on the oenologically beneficial traits were the most pronounced in *S. bacillaris*.

Regarding the nitrogen-related features of the *S. bacillaris* strains, namely the moderate yeast assimilable nitrogen demand and the noticeable ability to utilize proline at higher sugar level, this species could be a promising match in a mixed fermentation with *S. cerevisiae,* especially for grape juices with high or even extremely high sugar concentration.

In this experiment, the adequacy of this non-*Saccharomyces* species for winemaking, in combination with a suitable *Saccharomyces* yeast, is highlighted. Moreover, the results suggest that *S. bacillaris* possesses a more active adaptation mechanism to extreme sugar concentration. Undoubtedly, a more detailed investigation would be useful in the future focusing on the minor and volatile metabolites with an even wider strain set.
